# The outcasts, the sick, and the undead: atypical burials of the late medieval to modern greater Poland

**DOI:** 10.1038/s41598-025-04425-2

**Published:** 2025-06-04

**Authors:** Joanna H Bonczarowska, Joanna Wysocka, Beata Drupka, Nicolas Antonio da Silva, Ben Krause-Kyora, Marcin Krzepkowski

**Affiliations:** 1 Institute of Clinical Molecular Biology, Kiel University, Kiel, Germany; 2 Department of Genetics, Yale School of Medicine, New Haven, CT USA; 3 Department of Anthropology, Hirszfeld Institute of Immunology and Experimental Therapy, Polish Academy of Sciences, Wrocław, Poland; 4 Department of Anthropology, University of Wrocław, Wrocław, Poland; 5 Regional Museum of Wągrowiec, Wągrowiec, Poland

**Keywords:** Ancient DNA, Infectious disease, Plague, *Yersinia pestis*, Kinship, Undead, Molecular biology, Diseases

## Abstract

Past burial practices can provide insights into the social status of the inhumed and inform us of how the individuals were perceived by their community. Atypical or “deviant” burials may indicate that the deceased was considered an outcast, either due to disease, different provenance, criminal activity, or religious beliefs. To explore the importance of atypical burial practices in medieval and modern Poland, an interdisciplinary study was conducted on three Polish cemeteries (Dzwonowo, Skoki, and Wągrowiec) dating to the 14th -18th centuries CE. Twelve individuals were examined using archaeological evidence, anthropological analysis, and ancient DNA (aDNA) analysis. This study aimed to determine the underlying reasons behind the atypical nature of the burials (i.e., whether the individuals were outsiders, outcasts, relatives, or victims of infectious disease) and shed light on the complex interplay between health, social status, and community perception in medieval and modern Poland. The results revealed that a non-adult male and a middle-aged female, who were holding hands in a double burial in Skoki, possibly died from the plague. *Yersinia pestis* DNA was recovered from the non-adult individual, making it the third confirmed case of plague in Poland. Using the nonmetric skeletal traits, archaeological assemblage, and aDNA analysis, we identified several individuals as likely “outsiders” in the community. The results of this study expand our knowledge of past societal structures and cultural responses to disease as well as provide crucial context for interpreting burial patterns elsewhere in Europe.

**Supplementary Information:**

The online version contains supplementary material available at 10.1038/s41598-025-04425-2.

## Introduction

In medieval and modern Europe, one’s religious beliefs, provenance, health status, or even physical appearance could have formed the basis for social ostracism. The “outcast” status could also be carried by individuals who were considered to have been otherworldly creatures, criminals, or outsiders^1–4^. Often, the social status of the outcasts, the sick, and the strangers followed them into their graves in the form of a relatively atypical “deviant” burial, i.e., a burial that deviates from the standard practice at the time. Characteristics of such burials may include an unusual positioning of the body, the grave location within the cemetery, signs of violence or restraints on the remains, and multiple individuals buried together in one grave pit^3^. Double and mass graves found in necropolises from the late medieval (1250–1500 CE) and modern (1500–1795 CE) periods are often interpreted in two ways. Firstly, they might be an indication of a kinship or relationship between the individuals buried in them^5^. Secondly, they are viewed as evidence of violent occurrences, such as warfare, accidents, famines, or outbreaks of infectious diseases, which necessitate the burial of many people at once^6,7^.

Greater Poland (Polish: Wielkopolska) is a historically significant region of Poland, recognized as the birthplace of the Polish state in the 10 th century CE^8^. The name “Polonia Maior” (Latin for Greater Poland) was recorded as early as 1257^9^. This study examines atypical burials from cemeteries located at three archaeological sites (Skoki, Wągrowiec, and Dzwonowo) situated in Greater Poland. These cemeteries, dating from the 14th to 18th centuries CE, contain unusual burial practices that are the focus of this paper. When the examined cemeteries were in use, this region of Greater Poland belonged to the Polish Crown and the Polish-Lithuanian Commonwealth, with Catholicism being the dominant religion. At the time, bishops played a key role in society, including establishing burial rules^10^. The typical burial practices involved laying the deceased on their back, with the upper limbs positioned alongside the trunk or resting on the pelvis, either placed in a coffin or wrapped in shrouds, oriented in a W-E, with the head to the west (as Christian tradition compels)^11,12^. The location of the burial within the cemetery was closely linked to social status and wealth. The richest individuals and members of the clergy were interred within or near the church, while those of lesser importance were buried further away. Criminals, unbaptized children, those who died of “plague air”, and others considered unworthy or dangerous were frequently buried at the auxiliary cemeteries located on the cemetery outskirts or beyond their boundaries on unconsecrated grounds^10,12,13^) (see more in Supplementary materials).

Similar to other parts of Europe, infectious diseases had a noticeable impact on the population of Poland in the medieval and early modern periods. Greater Poland experienced frequent warfare, along with army marches and famines, which accelerated the spread of disease and led to a rise in fatalities. The most significant devastation and depopulation occurred during the Polish-Swedish War (1655–1660), commonly referred to as the Deluge^14^, and the Great Northern War (1700–1721). Further destruction was brought upon the Seven Years’ War (1756–1763)^15^. In addition, following the political turmoil that erupted in the country after previous conflicts, Poland experienced a complete partition by Prussia, Russia, and Austria in the late 18th century, resulting in Greater Poland coming under Prussian control. Consequently, Poland ceased to exist as an autonomous nation for 123 years^16^.

The conflicts were accompanied by outbreaks of epidemics, with the 1707–1710 CE plague epidemic being particularly tragic, with —approximately 65% of the total population (around 9,000 individuals) perishing during the outbreak in the capital of Greater Poland - Poznań^17^. The epidemics were also repeatedly recorded in the late medieval and modern Cistercian monastery town of Wągrowiec (14th-18th/19th c. CE)^18^. It is estimated that Wągrowiec could have lost up to half its population during that time due to the plague^18,19^. Closely neighboring settlements of Dzwonowo (14th–18th c. CE) and Skoki (15th–18th c. CE) were likely also impacted by such events, although no known written records of the epidemic’s impact on these towns exist. A cemetery discovered on the outskirts of Skoki seems to have served an auxiliary function to the main parish cemetery. As such burial grounds were often a place of inhumation for criminals and the victims of epidemics, it can be hypothesized that these “outcasts” might have been buried there^5^. Burials exhibiting visible apotropaic rituals, dating to the 14th−18th centuries, have been documented previously in close vicinity (up to 90 km) to the sites examined in this study. Such unusual practices included placing a sickle above a deceased’s neck, as found in Drawsko e.g.^4,20,21^, and Pień^22^, weighting down an individual with stones in Pień^23^ and symbolically immobilizing an individual with a padlock in Skoki and Pień^23,24^. Other atypical burials observed in Skoki and Pień included graves containing multiple individuals and were interpreted as a plague pit and burials of relatives, respectively^6,24,25^.

Due to the omnipresence of infectious diseases in medieval and modern Europe^26–29^, uncommon burial practices are often interpreted as suggestive of disease, as mass deaths linked to epidemics frequently resulted in mass- and multiple burials e.g^30,31^. Furthermore, different practices were applied to protect society from the spread of disease or peace disruption by the undead^6,7,32^. In some cases, certain illnesses can be determined based on paleopathological and/or ancient DNA (aDNA) analysis of skeletal remains [e.g^26,27,33^. While there are limitations to these methods^33,34^, they can be complementary to each other, providing a more comprehensive picture of health in past populations. For this reason, the examination of skeletal remains in the context of disease calls for a multidisciplinary approach.

The study aims to (1) examine the relationship between atypical burial practices in late medieval/modern Greater Poland and the occurrence of infectious diseases, including plague; (2) investigate the association between the unusual positioning of the body, uncommon grave goods, and the origins of the individuals; (3) evaluate the kinship between individuals buried together and explore the possibility of infectious disease as the cause of death. To fulfill the aims, we analyzed atypical graves through a combination of archaeological evidence, osteological examination, and aDNA analysis. With this approach, we provided insights into the potential reasons behind the unusual treatment of individuals after death, shedding light on the social dynamics in those communities and their treatment of the sick and the outsiders.

## Materials and methods

### Material

**Fig. 1:**
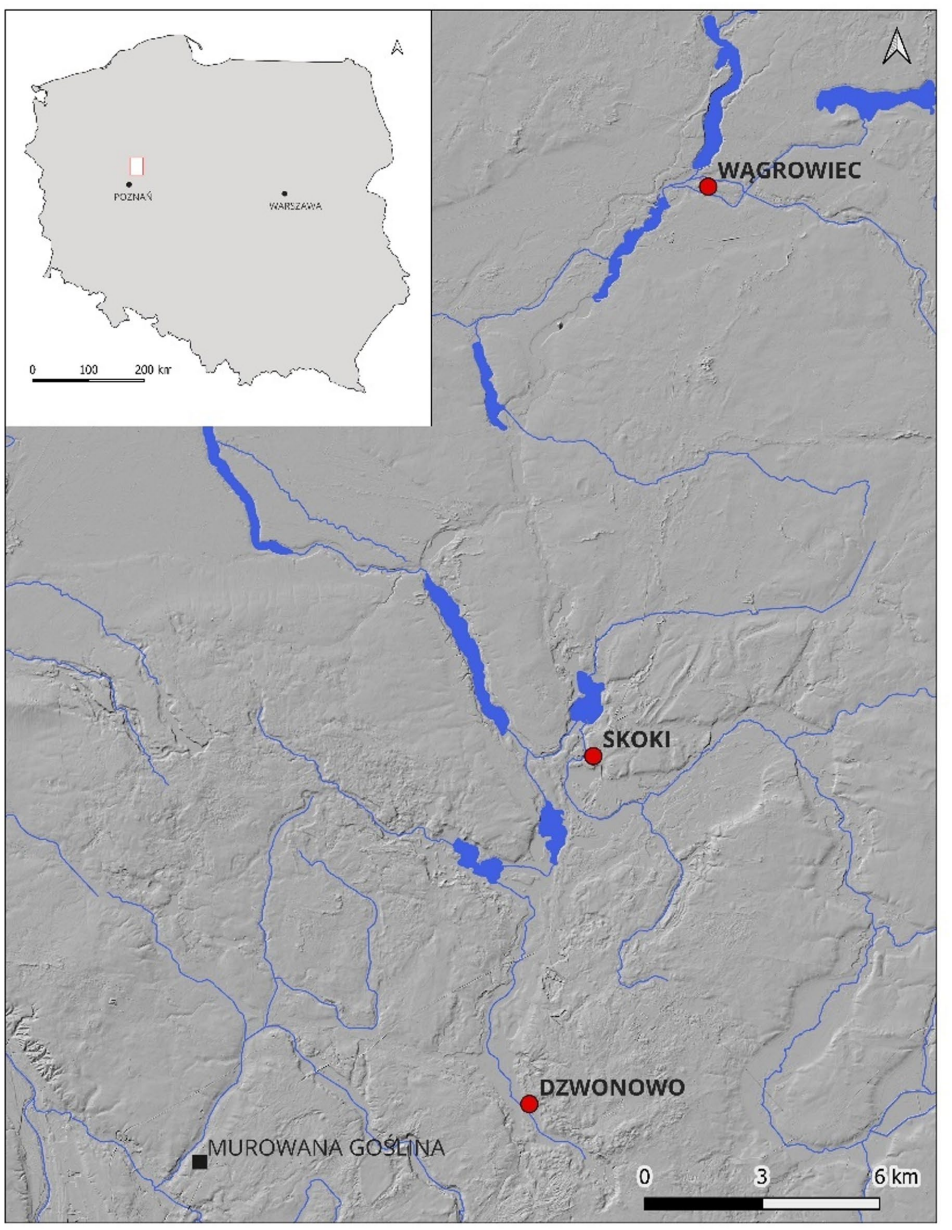
Archaeological sites examined in this study. A digital elevation model (DEM) with the location of cemeteries included in the study: Wągrowiec, church parish cemetery (late 1th−18th/19th c.CE); Skoki, suburban cemetery (15th–18th c. CE); Dzwonowo, the church cemetery of the city’s parish (early 14th century−2nd half of the 15th c.CE) and later the rural church cemetery (2nd half of the 15th century−2nd half of the 18th c. CE). (DEM was developed in an open-source program (QGIS 3.42 - https://qgis.org/download/) based on publicly available point cloud from airborne LIDAR laser scanning downloaded from the geoportal platform (gov.pl)).

In this study, three urban cemeteries were explored in the context of atypical burials. The sites include the Wągrowiec church parish cemetery (late 14th–18th/19th c. CE), the Skoki suburban cemetery (15th–18th c. CE), and the parish church cemetery in Dzwonowo (early 14th–late 15th c. CE) that became rural church cemetery when the town diminished into a village (late 15th–late 18th century) (see Supplementary Material 1 for more details). The sites were located within an approximately 30 km (~ 8 miles) radius of each other in Greater Poland (Wielkopolska) (Fig. 1). They were chosen for the study based on the recorded history of epidemics, army marches in the area^18,19^, and the auxiliary character of the cemetery in Skoki. The selection of individuals for this study was guided by direct observations during field research. Particular attention was paid to burials that deviated from the standards applicable in each necropolis, mainly the unusual position of the limbs, distinctive grave goods or objects, localization of the graves, and instances of multiple individuals being placed in a single burial pit (Table 1). In total, the remains of 12 individuals from 10 grave pits were selected for analysis. The material included eight individuals from Skoki, two from Wągrowiec, and two from Dzwonowo (Table 1).

**Fig. 2:**
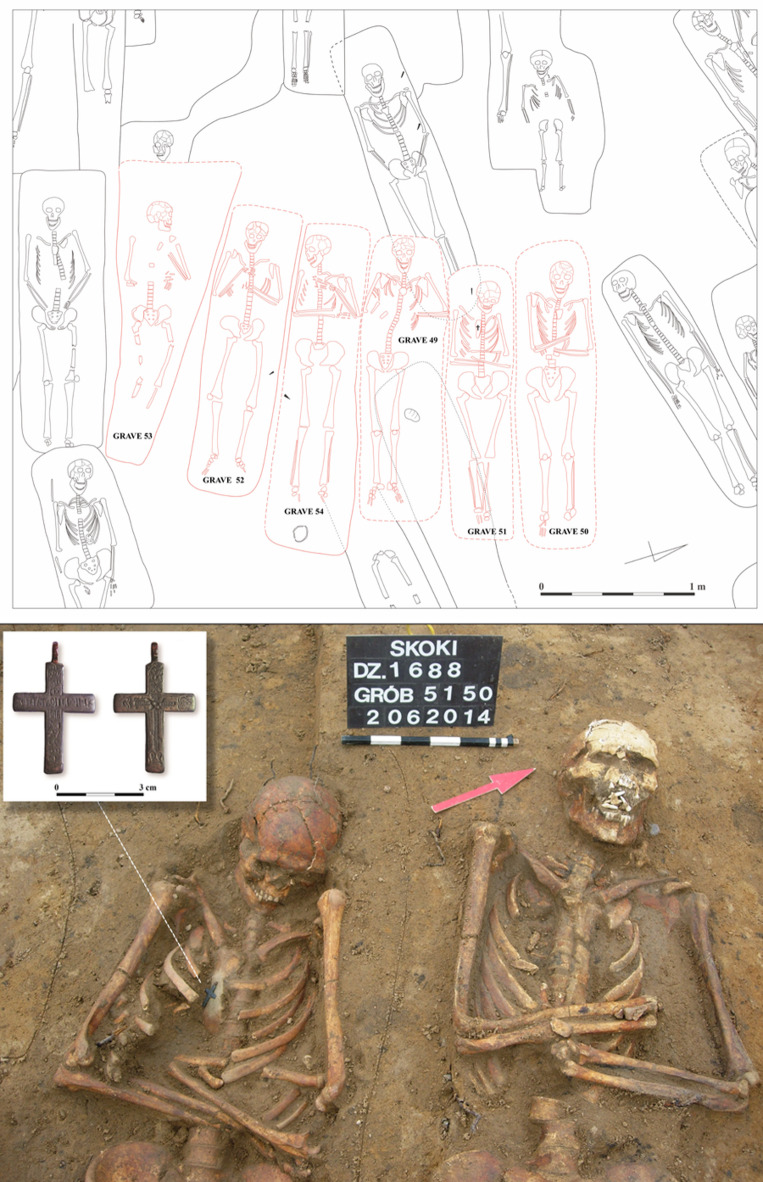
A single row of individuals with an atypical upper limbs position (in red S49-S54) interpreted as Russian soldiers (upper panel) alongside a close-up of two burials of (S50 and S51) where Orthodox crosses were found (lower panel).

**Fig. 3:**
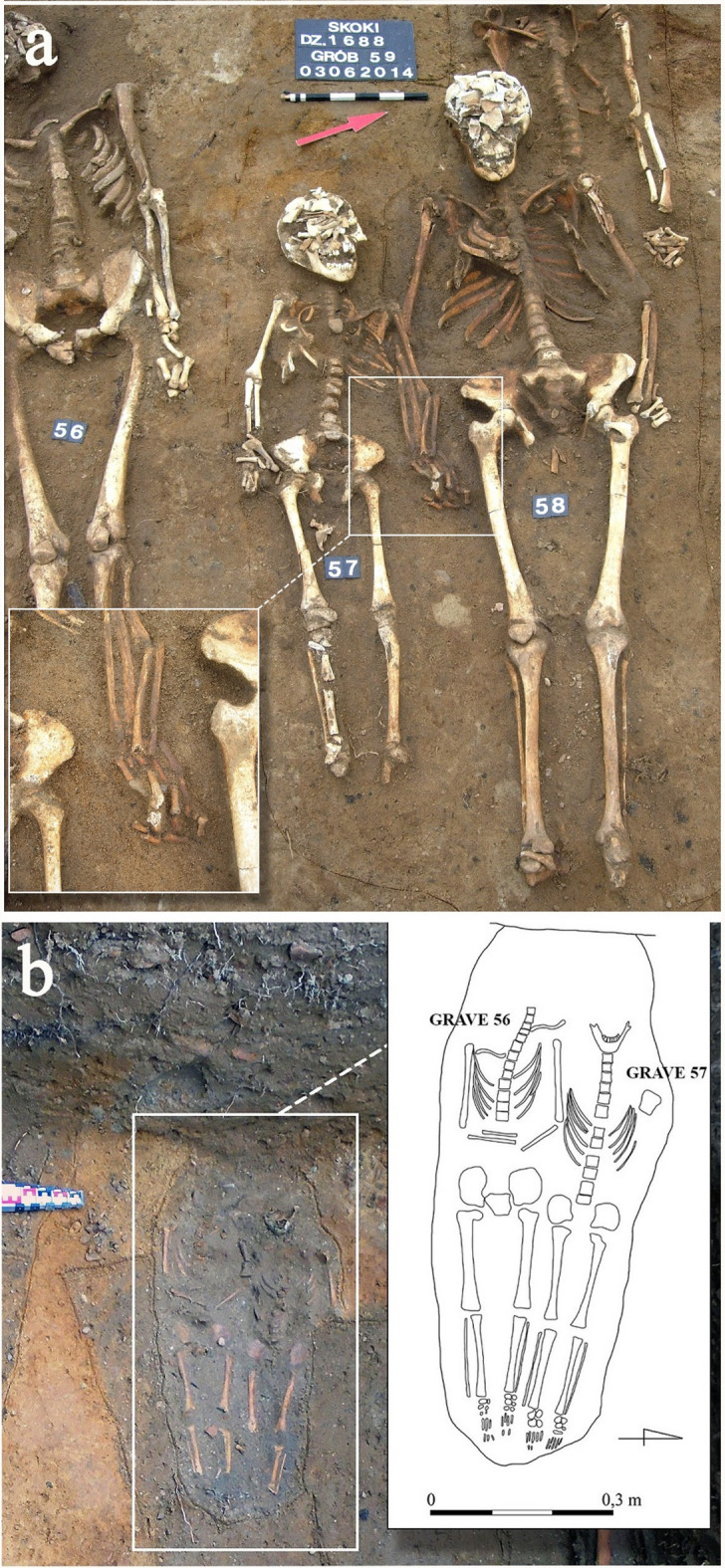
(**a**) A double burial of a non-adult (S57) and an adult (S58) holding hands excavated in Skoki; (**b**) A double burial of non-adults from Wągrowiec (W56 and W57).

The remains of six adult individuals (S49-54) buried within a short period in single graves were discovered forming a clear row^35^. The presence of characteristic Orthodox crosses (S50 and S51), along with the positioning of the upper limbs (Fig. 2), has led to the identification of the individuals as Russian soldiers. The soldiers are probably linked to the Great Northern War (1700–1721) or the Seven Years’ War (1756–1763). The arrangement of the upper limbs, crossed on the stomach or strongly bent and placed on the chest (Fig. 2), is typical of Orthodox cemeteries^36,37^ and was unusual for the necropolis in Skoki. Another grave selected for this study is a double burial of S57 (non-adult) and S58 (adult). At the fieldwork stage, it was considered a possible burial of a closely related pair, such as mother and child, who died at the same time, as indicated by their clasped hands (Fig. 3a). A double burial of two non-adult individuals (W56 and W57), possibly siblings, was also excavated in Wągrowiec (Fig. 3b). The burials selected from Dzwonowo (DZ22, DZ23) were located on the periphery of the cemetery, where the graves were arranged more loosely compared to the densely packed burials found in the central area. Additionally, DZ22 was buried directly below a stillborn 7-month-old fetus (not included in this study) positioned in a south-to-north orientation, perpendicular to other burials. The most probable reason for the unusual burial of the stillborn is that it died being unbaptized and thus, was inhumed on the periphery of the cemetery. This further highlights the atypical character of the burial ground. DZ22 and DZ23 were not only isolated from the other graves but also treated in a distinctive manner (see Table 1). Seemingly, special measures were taken to protect the living from potential infection or harmful influences from the deceased^5,38,39^, including weighting down the body with stones (DZ22), filling the coffin with a lime-like substance, and sealing it with an unusually high number of nails (n=49) (DZ23) (up to three nails were found in other graves at this cemetery).

**Table 1:** Atypical characteristics of the burials and individuals selected for this study.

Site	Dating (CE)	Grave *N*	Atypical characteristics of the burial
Skoki	15th–18th c.	S49	one row of single graves; (Fig. 2) upper limbs crossed over the stomach or chest – typical for Orthodox cemeteries^36,37^ Orthodox crosses (S50 and S51) suggesting the individuals were Russian soldiers (~ 1700–1763 CE) (Fig. 2) [35]
		S50	
		S51	
		S52	
		S53	
		S54	
		S57	double burial of an adult “holding hands” with a non-adult (Fig. 3a)
		S58	
Wągrowiec	15th–16th c.*	W56	double burial of non-adult individuals (Fig. 3b)
		W57	
Dzwonowo	14th–18th c.	DZ22	two stones (the larger was ~ 15 × 19 cm) found above the cervical vertebrae (Fig. 4A); located on the outskirts of the cemetery
		DZ23	rectangular coffin with remains of 49 iron nails and white substance (likely lime); located on the outskirts of the cemetery (Fig. 4B)

*Dating of the grave is based on the stratigraphic analysis (dating of the whole cemetery is 14th−18th/19th c. CE).

**Fig. 4:**
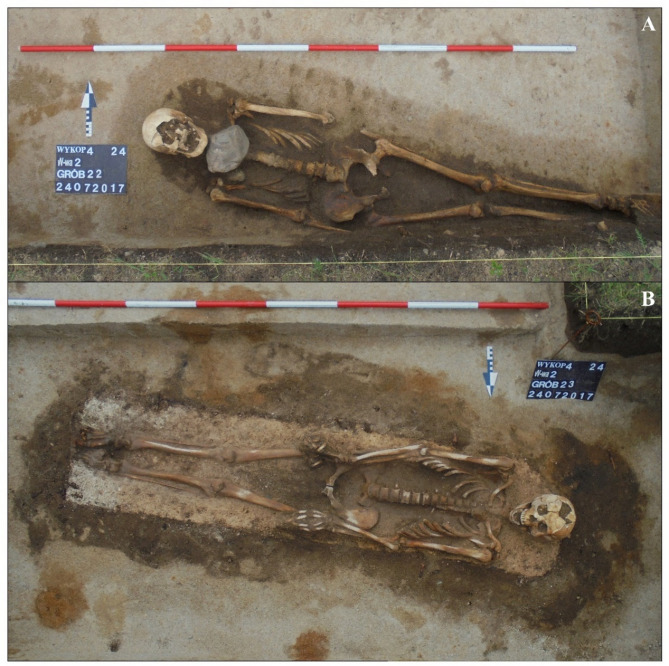
Atypical burials from Dzwonowo. Individual DZ22 buried with two stones above the cervical vertebrae (upper panel); Individual DZ23 covered with a white substance (lime) and buried in a rectangular coffin sealed with 49 iron nails (lower panel).

## Methods

The study was performed in accordance with regulations on research on human remains in Poland and Germany^40–42^ and following ethics guidelines for working with aDNA^43^.

### Osteological analysis

The biological profile of adult individuals was estimated based on the skull and pelvic features^44–47^. The age-at-death of non-adult individuals was assessed based on the tooth eruption^48^, the degree of the epiphyseal fusion^49^, and length measurements of the long bones’ shaft^50^. The skeletons underwent macroscopic analysis to identify and interpret trauma, including the mechanism of the injury and the type of fracture^51–53^. The time of the fracture was estimated using standard three levels of nomenclature: antemortem, perimortem, and postmortem^54^. Moreover, the skeletons were examined for the occurrence of lesions suggestive of infectious disease, including tuberculosis (inflammatory periosteal reactions and destructive inflammatory lesions on the visceral surface of the ribs, the shafts of the long bones, the vertebrae, and the pelvis)^33,55,56^. The inclusion criteria required that at least 50% of the skeleton be preserved, with completeness assessed according to the methods outlined by Rowbotham and colleagues^57^. Additionally, the presence of shovel-shaped incisors was documented^58^; individuals were excluded from this part of the study if half of their incisors were not preserved.

### Spectroscopic analysis

X-ray fluorescence (XRF) analysis was carried out to identify the composition of the white substance found in grave DZ23 and assess whether it was lime.

### DNA extraction, sequencing, and postprocessing

A total of 23 bones and teeth samples from 12 individuals (Table S1) were extracted and processed in a dedicated ancient DNA facility at the University of Kiel following the guidelines on contamination control in aDNA^59–61^. DNA extraction and library preparation were performed according to a previously published protocol for the half-UGD-treated samples^62^. Approximately 100 mg of bone/tooth powder was used for DNA extraction. The samples were treated with a digestion buffer (EDTA with Proteinase K) and incubated overnight at 37 °C with rotation. The lysates were then purified, and uracil residues were removed with USER, followed by enzyme inhibition after 30 min. The blunt DNA ends were repaired, and the samples were purified before adapter ligation. Following a double purification, and a fill-in step, the libraries were indexed through PCR amplification and purified. Shotgun sequencing was conducted using the Illumina HiSeq 6000 (2 × 75) platform at the Institute of Clinical Molecular Biology (IKMB) in Kiel, Germany. Following sequencing, the adapters were trimmed and paired-end reads were merged using ClipAndMerge v1.7.7^63^.

### Pathogen screening and authentication

Metagenomic datasets were screened for the presence of pathogen DNA with Megan Alignment Tool 0.3.0 (MALT)^64^. SemiGlobal alignment mode was used with a sequence identity threshold of 90%. A custom database consisting of all complete bacterial genomes from the NCBI website was used (download date: 24.01.2019). Screening results were visually inspected using the MEGAN software^65^.

To authenticate the parvovirus B19 findings, reads aligning with the B19 were extracted into a fasta file and blasted against the NCBI database with blastn (megablast, default parameters). *Yersinia pestis*-positive sample was mapped against the CO92 reference genome (NC_003143.1, NC003131.1, NC_003132.1, NC_003134.1,) with Burrows-Wheeler Aligner (BWA) v0.7.12 (*n* = 0.01, l = 1024)^66^. The output bam file was converted into a fastq with bedtools bamtofastq^67^ and aligned against *Y. pestis* CO92 and ten closely related *Yersinia* species (Table S2) in a competitive mapping, using the aln -n 0.01 option of the BWA aligner. Reads of quality below 30 were filtered out. Samtools idxstats (SAMtools v1.12)^68^ was used to examine the number of aligned reads.

### Contamination assessment and genotyping

Due to expected deamination, all reads were mapped to the human genome reference build hg19 using BWA v0.7.15^66^ with reduced stringency settings, as denoted by the flag -n 0.01. Duplicated reads were removed using DeDup v0.12.1^63^. To confirm the ancient origin of the sequences, terminal damage of the reads (C to T substitutions) was assessed with DamageProfiler^69^. After the validation, the first two positions from the 5’ and 3’-ends of the reads were trimmed. Furthermore, X-chromosome and mitochondrial DNA contamination were assessed with ANGSD and Schmutzi, respectively^70,71^.

Genotypes for all individuals were generated in a pseudo-haploid manner with SequenceTools v1.2.2 (https://github.com/stschiff/sequenceTools) based on 1,233,013 positions from the 1240 K^72–74^. Individuals for whom less than 20,000 positions were covered were excluded from the analysis.

### Determination of genetic sex, MtDNA, and Y chromosome haplogroups

The genetic sex of the analyzed individuals was determined based on the ratio of X chromosome-derived shotgun sequencing data to the autosomal coverage^75^. Only samples with more than 1,000 reads were considered for sex determination. Haplogroups were determined with HaploGrep 2^76^ and yHaplo^77^ for mtDNA and Y chromosome, respectively. For the mapping and base quality, athreshold of 20 was used. Only mtDNA haplogroups with a quality score above 0.8 were considered. For Y haplogroups, the presence of at least 10 derived alleles was used as a threshold to make a call.

### Population genetics and kinship analysis

The genotyped samples were integrated with the 1240 K and Human Origins (HO) reference panels from the Allen Ancient DNA Resource (AADR; v50.0.p1), which include previously published genotypes of ancient and modern individuals^78^. Analyzed individuals were projected onto 66 modern West Eurasian populations^79^ in a principal component analysis (PCA) using 597,573 SNPs from the merged HO dataset. The option „lsqproject” from smartpca was used to approximate the PCA projection with excessive missing data^80^. Shared genetic drift between individuals from Poland and the modern West-Eurasian populations used in the PCA was measured using outgroup *f3* statistics in the format f_3_ (analyzed population; test population, Mbuti)^79^. Unsupervised admixture analysis for nine individuals from this study and 1067 published individuals (Table S3) was conducted using ADMIXTURE v1.3.0 [81 with k-values ranging from 3 to 6, each evaluated with 10 bootstrap replicates.

Biological relatedness was estimated according to Fowler and colleagues^82^. Pairwise allelic mismatch rates in autosomal sites from the 1240k panel were calculated for each pair of analyzed individuals. The median mismatch rate expected for unrelated individuals, used to calculate the relatedness coefficient (r), was derived from published data comprising 85 medieval individuals (Table S4). The cutoff values for kinship degrees were annotated after Fowler and colleagues^82^.

## Results

### Individuals inhumed in atypical burials

#### Biological and genetic profiles

Computational analysis of DNA sequences showed a deamination pattern characteristic for aDNA for all samples (Figure S1, Table S5), confirming their ancient origin. Among the 12 individuals examined in this study, there were nine adults (eight males and one female) and three non-adults (two males and one female) (Table 2 and S5). Six individuals from Skoki (S49-S54) were estimated as young (20–34 years old) or middle adult males (35–39 years old). The individuals buried in a double grave (S57, S58) were estimated to be an older female (45–49 years old) and a non-adult (14–17 years old) male. Two young children, a boy and a girl (2–3 years old), were inhumed together in a double burial in Wągrowiec. The individuals from Dzwonowo were estimated as a middle adult male (35–44 years old – DZ22) and a young adult male (25–35 years old – DZ23). Spectroscopic analysis confirmed that the white substance that covered the remains of DZ23 was lime.

**Table 2:** Characteristics of individuals buried in atypical graves.

Site	IndN	Sex^1^	Sex^2^	Age-at-death [years]	mtDNA	Y chr
Skoki	S49	M	M	20–21	J1c3 g	CT
	S50	M	M	30–34	–	–
	S51	M	M	27–35	–	–
	S52	M	M	35–39	X2e2a1	–
	S53	M	M	25–34	H36	–
	S54	M	M	20–25	–	–
	S57	–	M	14–17	H13b1	–
	S58	F	F	45–49	V	*n/a*
Wągrowiec	W56	– –	F	2–3	–	*n/a*
	W57		M	2–3	T2a1a6	–
Dzwonowo	DZ22	M	M	35–44	–	–
	DZ23	M	M	25–35	U5b2b1b	J

*IndN* individual number, ^1^ sex estimated using osteological methods, ^2^ genetic sex, *mtDNA* mtDNA haplogroup, *Ychr* Y chromosome haplogroup.

Seven different mitochondrial DNA (mtDNA) haplogroups were determined for seven individuals (Table 2), all of which are common for today’s Europeans. Available data was insufficient to determine the Y haplogroups with satisfactory resolution.

Assessment of non-metric dental features revealed shovel-shaped incisors in three individuals from Skoki (S49, S50, and S54), all of whom were identified as Russian soldiers based on the archaeological context. The preservation of the individuals from Wągrowiec (W56 and W57) did not allow for the observation of non-metric traits of the incisors, thus they were excluded from this part of the analysis.

### Kinship

**Fig. 5:**
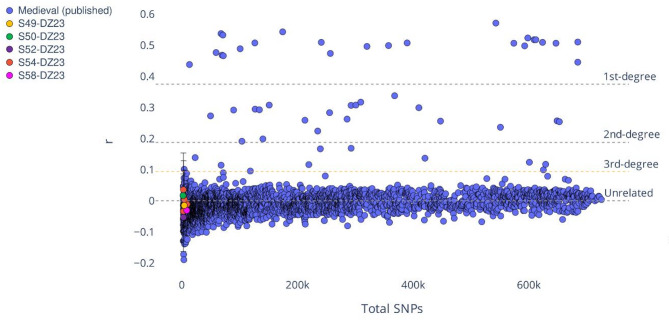
Results of the kinship analysis. Blue circles represent published medieval data (*n* = 85 individuals) that was used to calculate the median relatedness coefficient (r) for unrelated individuals. r was calculated according to the observed pairwise mismatch rate (see Methods). The dotted lines mark the r threshold for the corresponding degree of biological relatedness: 1 st degree, 2nd degree, and unrelated. Negative r values mean the pairs are less related than the average.

Biological relatedness was explored between the individuals buried together using the relatedness coefficient based on the pairwise allelic mismatch^80^ as well as the mitochondrial and Y chromosome haplogroups (as supporting evidence). Due to the low number of shared SNPs between individuals in pairwise comparisons (Figure S2), it was only possible to evaluate the relatedness of five individual pairs between DZ23 from Dzwonowo and five individuals from Skoki (S49, S50, S52, S54, and S58) (Fig. 5). The relatedness coefficient r was low for all five pairs and no kinship was detected in the analyzed sample. Distinct mtDNA haplogroups for the pair buried in a double grave from Skoki oppose the mother-son relationship.

### Population genetics

**Fig. 6:**
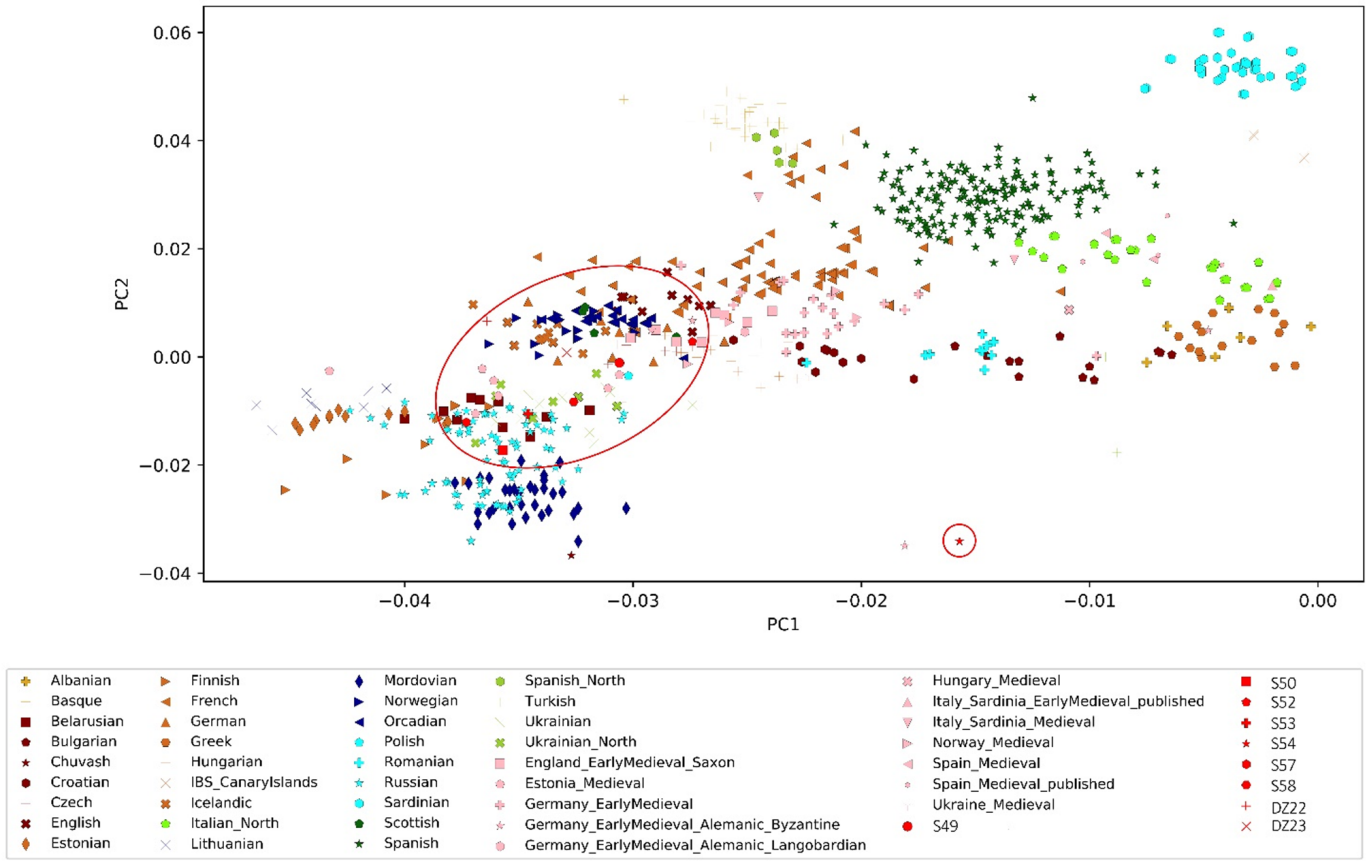
Principal component analysis of nine individuals from Skoki (*n* = 7) and Dzwonowo (*n* = 2) (marked in red) as well as 14 previously published medieval populations (marked in pink) were projected onto the first two principal components calculated for 66 present-day West Eurasian populations. For clearer visualization, only the most closely clustering 43 populations were plotted.

To investigate whether there is a link between the atypical burial practice and the origin of the individual, admixture and principal component analyses were performed. Individuals for whom fewer than 20,000 SNPs were called or/and contamination exceeded 5% were excluded from further analysis. In total, genetic data of nine individuals (from Skoki (*n* = 7) and Dzwonowo (*n* = 2)) was visualized together with previously published present-day West-Eurasian populations (*n* = 66) and medieval Europeans (*n* = 14) (Fig. 6).

In the PCA, two individuals from Dzwonowo and Skoki (S49 and S52) cluster among representatives of present-day Northern and Western European populations. Individuals S50, S53, S57, and S58 overlap the clusters of present-day Eastern Europeans, such as Ukrainians, Russians, and Belarusians, as well as medieval Estonians. S54 is markedly separated from the remaining individuals along both PC1 and PC2. This finding is also reflected in the results of admixture analysis (Figure S3 and S4), showing that S54 carried two main genetic components: one maximized in the present-day Finnish and the other in the Bedouin and Druze populations. S54 also lacked the component maximized in the Iberians, Basque, and French that was carried by other individuals from Skoki and Dzwonowo.

**Fig. 7:**
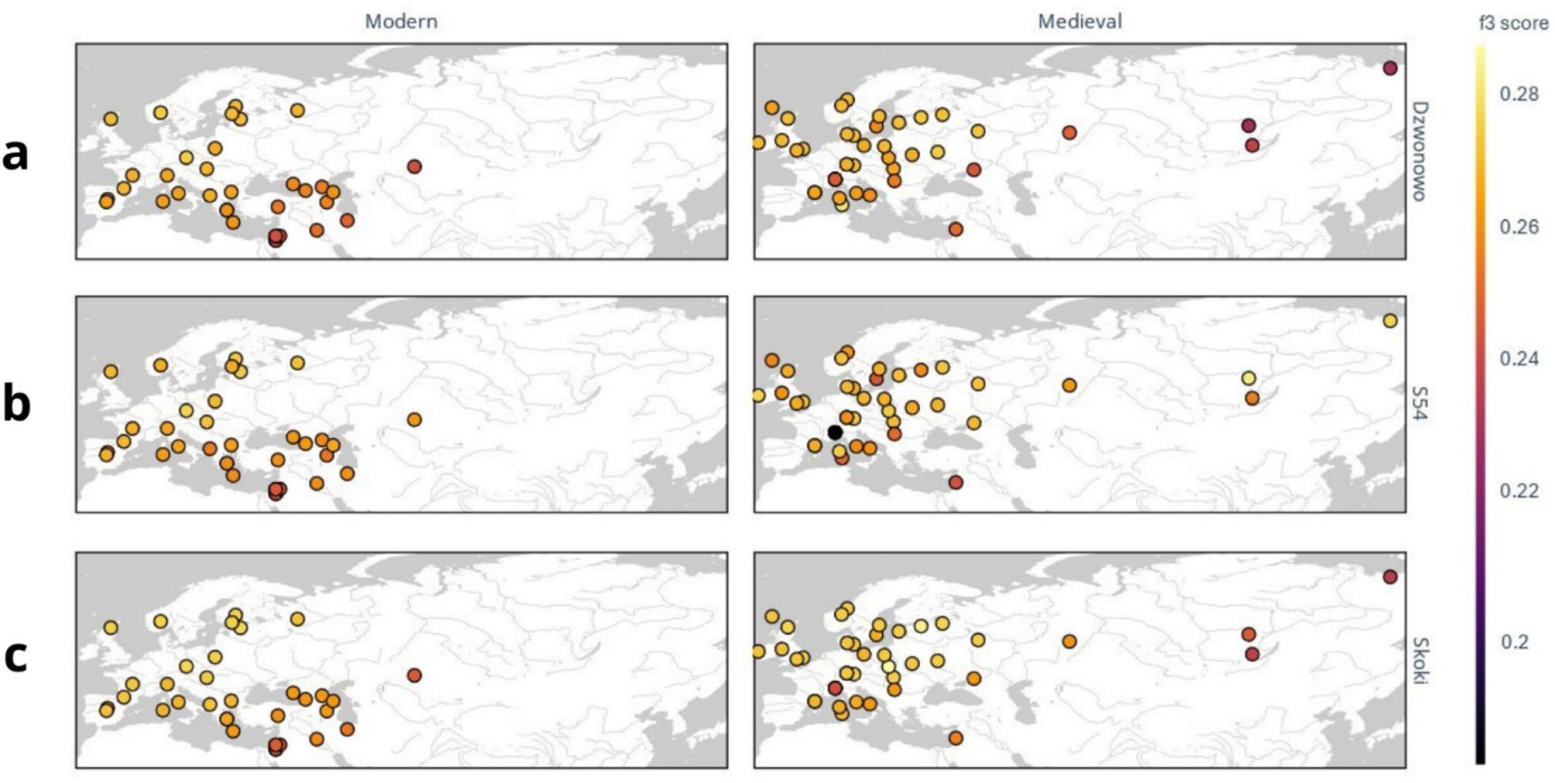
f3-outgroup statistics showing the amount of shared genetic drift between the individuals from Poland (**a**) Dzwonowo, (**b**) S54 and (**c**) Skoki and previously published present-day (modern) and medieval populations.

To further explore the genetic history of the individuals, the outgroup-f3 statistic was calculated separately for Dzwonowo, Skoki (excluding individual S54), and S54 separately (Fig. 7, Table S6). The analysis showed that the individuals from Dzwonowo and Skoki share the largest amount of genetic drift with populations of Central, Eastern, and Northern Europe (Fig. 7a,c). Results of the f3 statistics for S54 do not differ substantially when present-day populations are considered. However, relative to medieval populations, S54 shares the largest amount of genetic drift with individuals from the region of today’s Siberia (Fig. 7b).

### Osteological and molecular palaeopathology

The analysis of the traumatic injuries revealed numerous antemortem traumas within analyzed individuals with an accumulation of fractures within two individuals from Dzwonowo (DZ22 and DZ23). Among individuals from Skoki, only one (S50) exhibited traumatic bone lesions. Lesions indicative of traumatic injury were also observed in individual W57 from the double burial in Wągrowiec (Table 3). Excessive postmortem trauma of the bones was common among the material as the remains, especially from Skoki, were heavily fragmented. No inflammatory periosteal reactions or destructive lesions were observed within the material.

**Table 3:** Observed pathological conditions within the individuals from the three sites (Skoki, Dzwonowo, Wągrowiec).

IndN	Description of the pathological conditions	
S50	healed isolated fracture of the proximal part of the right ulna, remained severe porosity – Monteggia fracture fully healed vertebral compression fractures of fifth cervical and twelfth thoracic vertebrae (C5 and T12);	
DZ22	healed complete fractures of multiple ribs (six right (4 th-9 th), four left (6 th-8 th, 10 th), and five undetermined fragments):	
	left ribs (remained mild porosity): 6th – transverse midshaft fracture 7th – double transverse fractures: angle and midshaft (6 cm apart) 8th – transverse midshaft fracture 10th – double midshaft transverse fractures (3 cm apart)	right ribs (fully healed, no porosity): 4th and 5 h – oblique fracture of an angle 6th and 7 h – transverse midshaft fracture 8th – double midshaft transverse fractures (2 cm apart) 9th – double midshaft transverse fractures (5 cm apart)
	undetermined fragments (five): Transverse fractures of the midshaft (fully healed, no porosity)	
DZ23	healed complete fracture of multiple ribs (two right and seven left):	
	left ribs (fully healed, no porosity): 3rd – double transverse fractures: of the midshaft and sternal end 4th and 5th – transverse fracture of the sternal end 6th and 7th – transverse midshaft fracture 8th – transverse fracture of the sternal end 9th – oblique fracture of an angle and transverse fracture of the midshaft	right ribs: 7th – transverse fracture of the sternal end (fully healed, no porosity) 11th – transverse midshaft fracture (mild porosity)
	healed oblique fracture of the acromial end of the left clavicle, healed fracture of the spinous process of the first thoracic vertebra (T1);	
W57	healed fracture of the right clavicle (greenstick fracture localized on the shaft near the acromial end of the clavicle)	

*IndN* individual number.

To further test the hypothesis that atypical burial practices were connected to individuals suffering from infectious diseases, pathogen screening was performed. Molecular traces of pathogen DNA were detected in six individuals. Evidence of human parvovirus B19 infection was found at each of the cemeteries in a total of five individuals (S49, S51, W56, W57, and DZ23) (Table S5). B19 is a widespread virus best known for causing erythema infectiosum (fifth disease) in children. It is transmitted via respiratory droplets, blood, and vertically. The symptoms vary from mild to severe depending on the immunologic and hematologic status of the individual^83^. Moreover, DNA of *Yersinia pestis* (*Y. pestis*), the causative agent of plague, was detected in a tooth sample from an adolescent male (S57) from Skoki (Table 4).

To exclude the possibility that the *Y. pestis* sequences in the S57 sample were a result of environmental contamination, competitive mapping against *Y. pestis* and ten closely related *Yersinia* species was performed. The mapping and subsequent quality filtering revealed 82 *Y. pestis*-specific reads in the sample (Table 4), including nine reads aligning to the pPCP1 plasmid which is unique to the plague pathogen.

**Table 4:** Mapping characteristics of the S57 sequences against the CO92 *Y. pestis* reference genome. N of *Y. pestis-*specific reads was obtained via competitive mapping against the genomes of CO92 *Y. pestis* and ten closely related *Yersinia* (Table S2).

*N* of aligned reads (CO92)	Chromosome	pPCP1	pCD1	pMT1
unfiltered for MQ	3458	10	23	14
filtered for MQ ≥ 30	658	10	19	10
*Y. pestis*-specific	44	9	19	10

*MQ* mapping quality,* p* plasmid.

## Discussion

In this study, the atypical nature of ten burials from medieval and modern Greater Poland was explored using a multidisciplinary approach that combines archaeological, osteological, and molecular analyses. Our results point out several possible reasons behind atypical burial practices, such as different origins, beliefs, and diseases.

Adult male DZ22 from Dzwonowo was inhumed with two stones placed directly above the deceased (Fig. 3) In near proximity, another adult male DZ23 lay covered with lime. His coffin was closed with an unusually high number of iron nails (*n* = 49). The solidly made coffin and the use of lime in DZ23’s grave could be interpreted as protection necessary in the case of long-term transport of bodies. Such exports sporadically took place during the tragic epidemic of 1709, as reported in the census of the dead from the nearby Wągrowiec parish^18^ and could have been more frequent in between disease outbreaks. Furthermore, the use of lime cannot be ruled out as a protective measure against the spread of infectious diseases^84^. While paleopathological analysis did not reveal any signs of chronic infections, a molecular sign of infection with B19 was noted for DZ23. In immunocompromised individuals, B19 can cause persistent anemia. In those who are immunocompetent, the infection is usually asymptomatic or causes mild flu-like symptoms and body rash^83^. Interestingly, redness of the skin was considered as one of the indicators of being an “undead” (in Polish: “upiór” (singular) and “upiory” (plural))^32^. Despite the adoption of Christianity by Poland in 996 CE^85^, Polish culture was still full of superstitions in the post-medieval period. Various stories about the phantoms and the undead who rise from the dead to spread diseases as well as to attack people and cattle, were present in Polish folklore, poetry, ethnographic literature, and even press reports^32^. Those who suffered a violent death, i.e., during an epidemic, might have been perceived as the undead or even as the source of the epidemic outbreak^5,32,86^. This belief could arise either during the individual’s lifetime or shortly after their death and burial. The body of an individual suspected of having become an undead was treated with different care than others and special measures were applied that aimed at keeping the deceased in the grave and preventing any negative impacts they might have on the surviving members of the community^5,38,39^. Known practices included situating the body facing the ground, decapitating the body and placing the head between the feet, filling up the mouth and/or eyes of the deceased with poppy seeds, and piercing the body (all the limbs or only the heart) with an aspen stake^32^. The latter is still presented in the contemporary stories of pop-culture vampires. In this context, however, the use of the nomenclature “antivampire burials” is controversial, as there is no evidence of the use of the word “vampire” (Polish – wampir) in medieval^87^ or even early modern Poland^88^. Additionally, “upiór” is not the same creature as “wampir”, as an individual could have been recognized as “upiór” before death^32^ and thus the term was avoided throughout the paper. Nevertheless, it is possible that the stones, excessive number of iron nails, and the lime were supposed to keep DZ22 and DZ23 in their graves, preventing them from disrupting the peace of the living. In addition to the atypical nature of their burials, numerous traumatic changes, which were unusual within individuals buried in Dzwonowo, were observed for both individuals. These included healed broken chest bones and upper limbs (Table 3). The analysis of DZ23’s injuries suggests that he experienced at least two separate traumatic incidents. The clavicle fracture points to an indirect mechanism of injury and suggests a fall as a possible cause, which could also explain the broken vertebra and ribs^53^. The second event could have involved another fall or an instance of interpersonal violence later in his life, as demonstrated by the incomplete remodeling of the fracture in the eleventh right rib. Similarly, individual DZ22 sustained over 20 fractures across multiple bones, which can be attributed to at least two distinct events, as evidenced by the varying healing states of the left and right ribs. Like DZ23, these injuries could have resulted from either accidents or interpersonal violence. The presence of multiple fractures may indicate that the two individuals from Dzwonowo suffered from possible ostracism or incited fear within the community. Additionally, both males were buried at the outskirts of the cemetery along fetal remains oriented contrary to Christian traditions (which suggests an unbaptized stillborn individual^13^. The location of the remains within the cemetery, multiple injuries, and the atypical nature of the burials could suggest that these individuals were seen as unwanted members of society^2^. Although the reasons behind the burial practices applied for individuals DZ22 and DZ23 remain speculative, the unusual characteristics of the graves point to the possible “outcast status” of these individuals.

In some cases, individuals were, and still are, buried together due to kinship, emotional and social bonds, or marital relationships^5^. While the mother-son relationship hypothesized between an adult female S57 “holding hands” with a male non-adult S58 (Fig. 3a) was ruled out, other relationships, not necessarily blood-related, could explain the double burial. For the pair of young children from Wągrowiec (W56 and W57, Fig. 3b), the amount of genetic data was insufficient to resolve the question of kinship. Another common reason for interring multiple individuals into one grave pit is the sudden death of numerous individuals due to conflict or an epidemic event^6,7,30,31^. Paleopathological analysis revealed a healed clavicle fracture in W57. In newborns, greenstick fractures of the clavicle can arise because of complications during childbirth. They are frequently associated with the application of excessive force when a child is lodged in the birth canal^51^, thus the trauma observed in W57 was interpreted as a case of this nature. No lesions suggestive of a chronic yet deadly infection, such as tuberculosis, were observed for any of the individuals. Interestingly, both W56 and W57 were infected with B19. Although usually symptoms of B19 infection are mild, severe complications, such as meningitis, anemia, or aplastic crisis, can occur in individuals whose immunity is weakened. B19 infection is symptomatic mostly in children due to their immature immune system and the first-time exposure^83^. Nevertheless, based on the molecular findings alone, it is impossible to determine the cause of death for these two children. On the contrary, pathogen screening of the double graves in Skoki revealed *Y. pestis* DNA in a tooth sample belonging to the juvenile male S57 from Skoki, which was possibly the cause of death for the pair (S57 and S58). Although no trace of the pathogen was found in S58, the positive status of S57 could suggest that both individuals died as a result of the plague. The lack of *Y. pestis* DNA in the samples isolated from S58 could be explained by the issue of ancient DNA preservation. Previous molecular screenings of skeletal remains from plague mass burials resulted in relatively few positive cases^89–91^, illustrating the difficulty of *Y. pestis* detection. Cemeteries in Skoki and Wągrowiec were in use during the period of the medieval and post-medieval plague epidemics in this region. In 1709–1710, the plague epidemic was registered for the Wągrowiec parish^18^. The first wave of the plague epidemic claimed 567 lives, and the second wave killed another 300 people. It is estimated that the town could have lost up to half its population at the time^18,19^. Based on written records, it is known that the victims were buried in the cemetery on the outskirt of Bielawa, parish cemetery and the church’s necropolis, as well as under roadside crosses, in the gardens of townspeople, near houses, and in forests. Given the proximity of Skoki to Wągrowiec, which suffered from plague epidemics^18^, and our findings of *Y. pestis* in S57, it can be concluded that the outbreaks also reached the neighboring towns and smaller surrounding villages for which no known written reports exist. While *Y. pestis* has been detected in archaeological material from across Europe several times^94–96^, there were only two other reports of *Y. pestis* detection in Poland; in one individual from the Bronze Age^95^, and within human and rat skeletal remains from the 15th century CE Gdańsk^96^. Our findings of *Y. pestis* DNA in S57 are, therefore, just the third molecular evidence of plague in Poland and the only evidence of plague in Skoki.

Atypical burial practices may also provide insights into the person’s provenance or religious beliefs. The personal goods and the atypical position of the upper limbs in individuals from Skoki (S49-S54) allowed for their identification as orthodox Russian soldiers from the Great Northern War (1700–1721) or the Seven Years’ War (1756–1763) (Fig. 2)^35^. The Eastern European ancestry of several individuals buried at the Skoki cemetery was also reflected in the population genetics analysis (Figs. 6 and 7). Moreover, the Monteggia fracture of the right ulna observed in S50 (not accompanied by any fracture of the radius of the same side) resulted from direct force^53^ and aligned with an interpretation that he may have had a background as a soldier, as this type of injury is often associated with the act of parrying an attack or falling against sharp edge^53^. Furthermore, shovel-shaped incisors were observed in three of those individuals (S49, S50, and S54). Certain dental non-metric traits differ in frequencies among populations and allow for successful estimation of population affinity in big samples^97,98^. In the literature, shovel-shaped upper incisors are known to be more frequent in Asian and Asian-derived populations^97,99^.

This study provides valuable insights into the atypical burial practices in late medieval and modern Greater Poland. The combination of osteological analysis, aDNA screening, and archaeological evidence revealed complex social and biological narratives, including instances of trauma, disease, and outsider status within these communities. It is, however, imperative to mention the key limitations of the study. Firstly, the sample size examined here is relatively small, restricting broader generalizations regarding atypical burial practices at the time. Additionally, DNA preservation varied across individuals, limiting the insight into kinship and, potentially, infectious disease patterns. Future research should incorporate a larger dataset across multiple sites and include stable isotope analysis, which could further clarify the mobility and social standing of these individuals.

## Conclusion

Our findings revealed that the adolescent (S57) buried with the middle-aged female (S58) in a double grave at the Skoki cemetery was infected with *Y. pestis*, possibly a victim of a plague outbreak. While aDNA analysis did not confirm S58 as infected, both may have succumbed to the disease simultaneously. Despite no genetic relationship, their burial “holding hands” suggests a close relationship. Pathogen screening also identified parvovirus B19 in five individuals (S49, S51, W56-57, DZ23). At Dzwonowo, two individuals buried on the cemetery outskirts had notable traits possibly linked to shared accidents or violence. Their atypical burials may reflect social fears. Additionally, archaeological and genetic evidence suggests six individuals from Skoki (S49-S54) were likely Russian soldiers from the Great Northern War (1700–1721) or the Seven Years’ War (1756–1763). These findings offer valuable insights into the social dynamics and health conditions of late medieval and modern Greater Poland.

This study highlights the role of infectious diseases, individual provenance, and religious beliefs in shaping burial practices, emphasizing the need for interdisciplinary approaches to fully understand the factors behind the treatment of the deceased in past populations.

## Electronic supplementary material

Below is the link to the electronic supplementary material.

Supplementary Material 1

Supplementary Material 2

Supplementary Material 3

Supplementary Material 4

## Data Availability

Analyzed sequences are available through the European Nucleotide Archive under Accession Number PRJEB83228: http://www.ebi.ac.uk/ena/browser/view/< accession>.
