# Supplementary material for: The outcasts, the sick, and the undead: atypical burials of the late medieval to modern greater Poland
Source: Sci Rep. 2025 Jun 4;15:19608. doi: 10.1038/s41598-025-04425-2 (PMC12137733; doi:10.1038/s41598-025-04425-2)
Supplement: Supplementary file 2 — Supplementary Material 2 [file 41598_2025_4425_MOESM2_ESM.docx]

**The outcasts, the sick, and the undead**

**– Atypical burials of the late medieval/modern Greater Poland**

**Supplementary Figures**


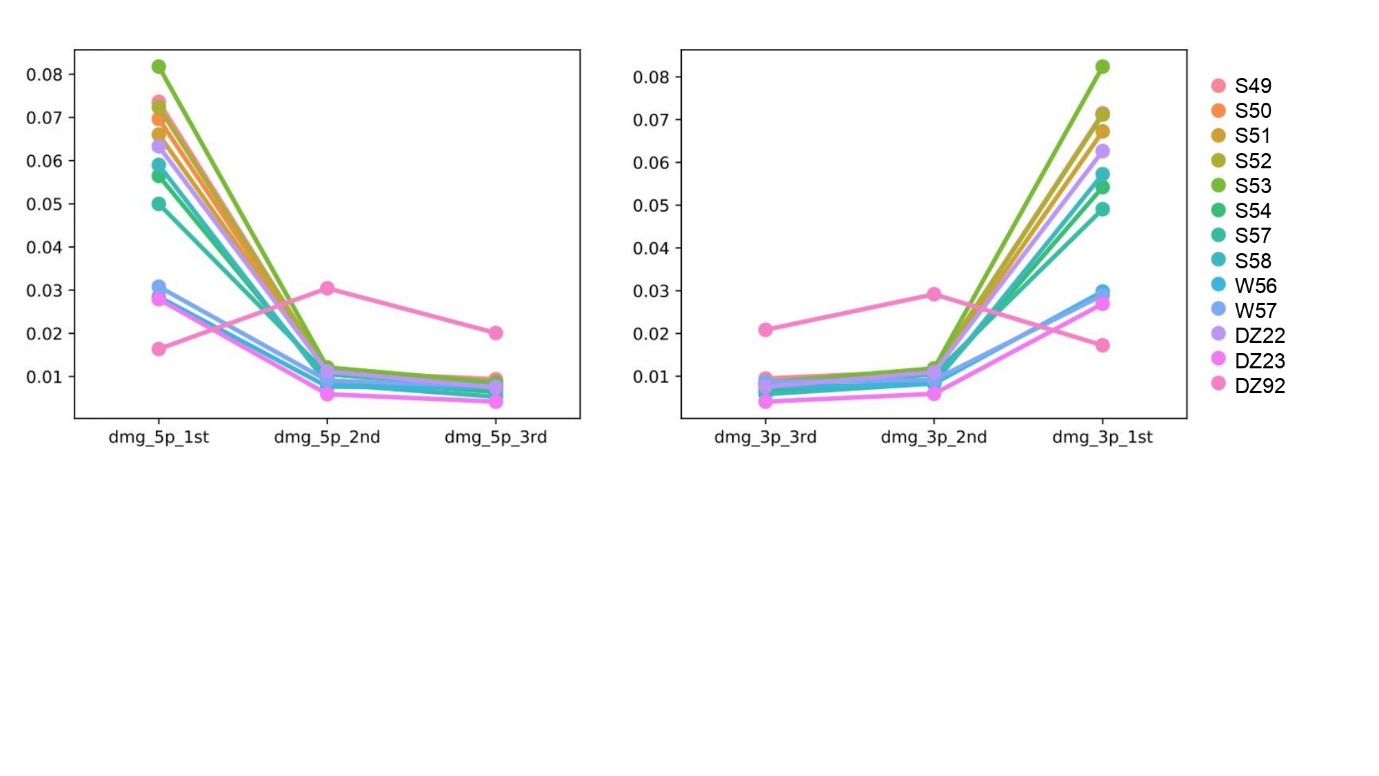


**Figure S1.** C to T substitution rates [%] (y axis) at the first three positions at the 5’ and 3’- ends (x axis) for the sequence reads of all individuals.

**Figure S2.** Observed pairwise mismatch rate that was used to calculate the relatedness coefficient r. Each marker represents one pairwise comparison. The x-axis show the number of shared SNPs between each pair.

**Figure S3.** Results from unsupervised admixture. The analysis was conducted using three to six components (K) across selected medieval and modern populations/individuals (total of 1067 individuals).

External pdf file


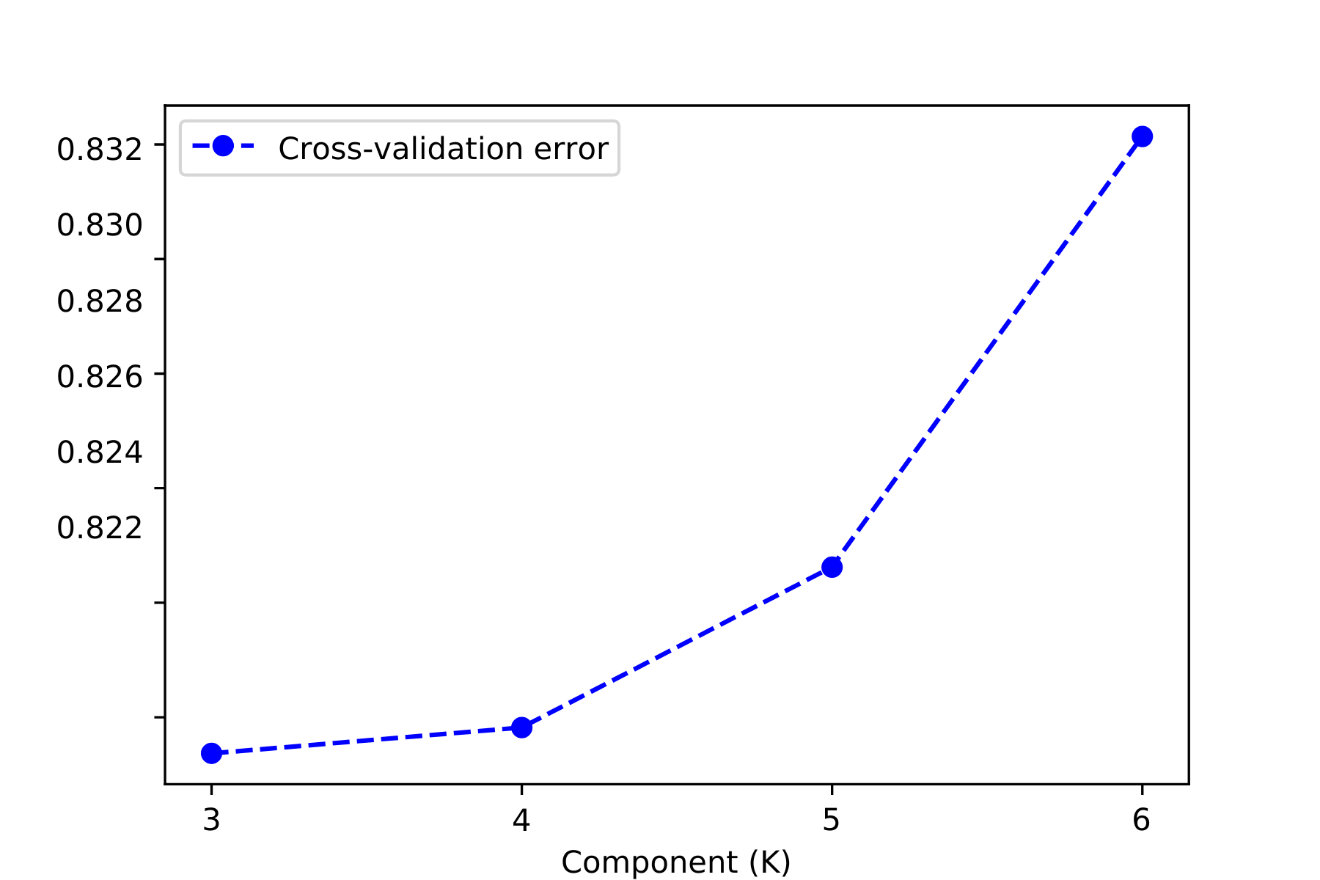


**Figure S4**. Cross-validation error for unsupervised admixture analysis. The cross-validation error was computed for each value of K, where K represents the number of components modeled. A lower cross-validation error indicates a better fit to the data.
