# Supplementary material for: The outcasts, the sick, and the undead: atypical burials of the late medieval to modern greater Poland
Source: Sci Rep. 2025 Jun 4;15:19608. doi: 10.1038/s41598-025-04425-2 (PMC12137733; doi:10.1038/s41598-025-04425-2)
Supplement: Supplementary file 3 — Supplementary Material 3 [file 41598_2025_4425_MOESM3_ESM.docx]

**The outcasts, the sick, and the undead – Atypical burials of the late medieval/modern Greater Poland**

**Authors:** Joanna H. Bonczarowska, Joanna Wysocka, Beata Drupka, Nicolas Antonio da Silva, Ben Krause-Kyora, Marcin Krzepkowski

**Supplementary Material 1**

**Skoki – suburban cemetery**

The cemetery was discovered on the outskirts of Skoki - a private town founded in 1367. Based on the coins discovered in the grave pits, it was established that this cemetery operated from the 15th to the 18th century, probably outside the development zone, serving an auxiliary function to the necropolis surrounding the parish church dedicated to Saint Nicholas Bishop. The origins of many areas of this type were related to the temporary burial places of epidemic victims and sometimes convicts. By their nature, suburban necropolises had a bad reputation and served people whose social or financial position made it impossible to be buried in a more dignified place (Duma, 2015). Therefore, poorer inhabitants of the suburbs, residents of hospitals and shelters, visitors, convicts, victims of war, soldiers, dissenters, suicides, actors, and others who were denied a place in the parish cemetery were buried there.

Mentions of such lower-class cemeteries, often not even dedicated, rarely appear in written and cartographic sources (Pietrzak et al., 2018). Information about burials taking place in the suburb of Skoki is not found in the fragmentarily preserved lists of the dead (*Liber Mortuorum*) of the local parish from 1690-1729, nor is it marked on any of the 19th-century maps. What is worth emphasizing, however, is that the memory of this necropolis has survived in the local tradition, and the place marked with a wooden cross functioned in the inhabitants' minds as a cholera cemetery. It can be assumed that it was used more intensively in 1565-1645 when the parish church was taken from the Catholics and handed over to the Czech brothers.

During archaeological rescue research, 118 burials were examined, and a large percentage of the deceased were equipped with coins, devotional items, and a spherical iron padlock (Krzepkowski et al., 2015). The largest group of graves that deviated from the standards was also recorded in this necropolis, some of which were included in this study.

**Wągrowiec – cemetery of the parish of St. Saint James the Apostle**

In the vicinity of Wągrowiec - a monastery town belonging to the Cistercians, epidemics were recorded, among others, in the years 1439, 1441, 1515, 1572, 1654, 1658, 1660, 1709 (Moeglich & Szymański, 2021). The demographic crisis, combined with subsequent natural disasters, caused a severe economic regression of the center, reflected in written sources several decades later (Krzepkowski & Moeglich, 2013; Moeglich & Szymański 2021). The invaluable source, which is the recently published daily list of people who died of the plague in the Wągrowiec municipal parish, makes it possible to reconstruct not only the dynamics of the course of this epidemic but also to trace the places where its victims were buried (Moeglich & Szymański 2021). The dead were buried in the church parish cemetery and the church's necropolis. Cemetery of All Saints in the suburbs of Bielawa were used during previous epidemics. Analysis of the census shows that the deceased were buried individually, and only in a few cases the data indirectly indicated the burial of two people in one grave pit. However, there is no information about burials in larger mass graves discovered in recent years in large urban centers of Central Europe, such as Gdańsk (Targ Sienny, Plac Dominikański) or the Czech Kutna Hora (Pudło, 2012; Kurkowska & Krzywdziński, 2016; Frolik, 2017, 2018).

The parish church in Wągrowiec, with its cemetery, was first mentioned in 1381. The necropolis operated until the turn of the 18th and 19th centuries when a new burial place was established on the eastern outskirts of the city (in place of an older necropolis used during periods of epidemics). During rescue research 66 graves were documented, including a double burial of non-adult individuals (W56-W57). Based on stratigraphic relations, the double burial was dated to the end of the late Middle Ages - the beginning of the modern era (15th-16th centuries).

**Dzwonowo – cemetery of the parish of St. Saint Michael the Archangel**

The cemetery was discovered in 2014 during archaeological surface research of the disappeared medieval town of Dzwonowo (*Zwanow, Swanowo*). Although the first historical mention of this temple comes only from 1348, it should be assumed that the parish was established shortly after the founding of the town, most likely back in the early 14th century. In the second half of the 15th century, the center fell and lost its status as a town, transforming into a village. The local church also fell into decline, losing its parish functions. However, the dead were still buried in its necropolis until the second half of the 17th century, when the ruined temple was finally demolished. During survey research carried out in the 2016 and 2017 seasons, 102 skeletal graves located at several levels and multiple coins dated until the 18^th^ century were discovered.

**References:**

1. Duma P. Śmierć nieczysta na Śląsku. Studia nad obrządkiem pogrzebowym społeczeństwa przedindustrialnego. Wrocław; 2015.
2. Pietrzak J, Obtułowicz Ł, Głąb H, Wróbel J. Odrzuceni przez społeczeństwo. Cmentarz z końca XV/1. połowy XVI wieku odkryty przy ulicy dworcowej w Gliwicach. Gliwice; 2018.
3. Krzepkowski M, Sikorski A, Wrzesińska A. Siedemnastowieczny pochówek zbiorowy z kłódką z cmentarzyska w Skokach, pow. wągrowiecki, woj. wielkopolskie. Próba interpretacji. In: Michałowski A, Teska M, Żółkiewski M, editors. Viator per devia scientiae itinera. Studia nad problematyką okresów przedrzymskiego, rzymskiego, wędrówek ludów i wczesnego średniowiecza. Poznań; 2015. pp. 339-53.
4. Krzepkowski M, Siłakowska M, Wrzesińska A. Osiemnastowieczne (?) pochówki żołnierzy rosyjskich ze Skoków, pow. wągrowiecki. Przykład identyfikacji „obcych” na późnośredniowieczno-nowożytnej nekropolii. In: Nowaczewska W, Bohr M, Drupka B, Wysocka J, Słodka A, editors. Możliwości badawcze w antropologii i archeologii. Wrocław; 2017. pp. 47-59.
5. Karczewska M, Karczewski M. Cmentarz wielowyznaniowy na wzgórzu św. Marii Magdaleny w Białymstoku. Historia miejsca. Białystok-Poznań; 2012.
6. Moeglich M, Szymański M. Taniec czarnej śmierci. Epidemie w dawnym Wągrowcu. Wągrowiec; 2021.
7. Krzepkowski M, Moeglich M. Kondycja gospodarcza miasta Wągrowca w czasach nowożytnych – dwa niepublikowane źródła z 1611 i 1739 r. Wągrowieckie Studia Muzealne; 2013.
8. Frolík J. Záchranný archeologický výzkum při odvodnění hřbitovního kostela Všech svatých s kostnicí v Kutné Hoře – Sedlci. Zprávy ČAS – Supplément; 2017. 105:34-5.
9. Frolík J. Pokračování záchranného archeologického výzkumu u hřbitovního kostela Všech svatých s kostnicí v Kutné Hoře – Sedlci. Zprávy ČAS – Supplément; 2018. 109:35-6.
10. Pudło A. Pochówek zbiorowy jako przykład grobu w czasie epidemii. In: Kizik E, editor. Dżuma, ospa, cholera. W trzechestną rocznicę wielkiej epidemii w Gdańsku i na ziemiach Rzeczypospolitej w latach 1708-1711. Materiały z konferencji naukowej zorganizowanej przez Muzeum Historyczne Miasta Gdańska i Instytut Historii PAN w dniach 21-22 maja 2009 roku. Gdańsk: Muzeum Historyczne Miasta Gdańska; 2012. p. 237–43.
11. Kurkowska K, Krzywdziński R, editors. Targ Sienny w Gdańsku – przedmieście europejskiej metropolii – w świetle wykopalisk archeologicznych. Katalog wystawy prezentowanej w Ratuszu Głównego Miasta w Gdańsku w okresie od 1 czerwca do 25 września 2016 roku. Gdańsk: Muzeum Historyczne Miasta Gdańska; 2016.
