# Supplementary material for: The outcasts, the sick, and the undead: atypical burials of the late medieval to modern greater Poland
Source: Sci Rep. 2025 Jun 4;15:19608. doi: 10.1038/s41598-025-04425-2 (PMC12137733; doi:10.1038/s41598-025-04425-2)
Supplement: Supplementary file 1 — Supplementary Material 1 [file 41598_2025_4425_MOESM1_ESM.pdf]

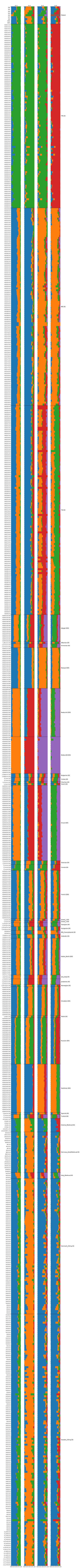
Poland
FIN.SG
IBS.SG
TSI.SG
Adygei.SDG
Albanian.DG
Armenian.DG
Basque.SDG
BedouinA.SDG
BedouinB.SDG
Bulgarian.DG
Cretan.DG
Chechen.DG
Czech.DG
Druze.SDG
Estonian.DG
Finnish.DG
French.SDG
Greek_1.DG
Greek_2.DG
Georgian.DG
Hungarian.DG
IBS_CanaryIslands.SG
Icelandic.DG
Italian_North.SDG
Jew_Iraqi.DG
Jordanian.DG
Norwegian.DG
Orcadian.SDG
Polish.DG
Russian.SDG
Sardinian.SDG
Spanish.DG
Turkish.DG
Estonia_Medieval.SG
Germany_EarlyMedieval.SG
Italy_Medieval.SG
Denmark_Viking.SG
Sweden_Viking.SG
